# A Novel Technique for Conduction System Pacing in Patients Undergoing Simultaneous Ablation of the Atrioventricular Node Using Axillary Venous Access

**DOI:** 10.19102/icrm.2025.16083

**Published:** 2025-08-15

**Authors:** Hussam Abuissa, Ahmed Elawad

**Affiliations:** Department of Cardiology, University School of Medicine, Omaha, NE, USA

**Keywords:** Ablation, atrioventricular node, axillary vein, conduction system pacing, His bundle

## Abstract

Conduction system pacing has emerged as a new pacing technique to achieve cardiac physiologic pacing, but its utility and safety in patients with atrial fibrillation undergoing simultaneous ablation of the atrioventricular node remains seemingly unknown. Here, we present a case series of 10 patients with long-standing persistent or permanent atrial fibrillation who failed rate-control therapy and elected to proceed with simultaneous ablation of the atrioventricular node and His-bundle pacemaker implantation.

## Introduction

Conduction system pacing has emerged as a new pacing technique to achieve cardiac physiologic pacing. However, its utility and safety in patients with atrial fibrillation undergoing simultaneous ablation of the atrioventricular node has not been previously studied, to the best of our knowledge. Here, we present a case series of 10 patients with long-standing persistent or permanent atrial fibrillation who failed rate-control therapy and elected to proceed with simultaneous ablation of the atrioventricular node and His-bundle pacemaker implantation, which we were able to achieve via an axillary approach with backup right ventricular apical pacing.

## Case series

We included 10 patients in this initial case series and have summarized their characteristics in **Table 1**. All patients had long-standing persistent or permanent atrial fibrillation with failure of rate-control therapy despite high doses of atrioventricular nodal blocking agents. Patients’ average age was about 75 years, and the majority were women. The left ventricular systolic function was mostly preserved, and none of the patients had an indication for implantable cardioverter-defibrillator or cardiac resynchronization therapy. Comorbidities included hypertension, type 2 diabetes mellitus, coronary artery disease, congestive heart failure (mostly diastolic), chronic kidney disease, and valvular heart disease. Interestingly, only one patient had baseline conduction system disease—namely, right bundle branch block. All patients were discharged home on the same day and had no immediate periprocedural complications. They were followed up for about 2 years with a minimum follow-up period of 1 year. Remote monitoring was performed every 3 months as per our, protocol with no significant increase in the His-bundle lead pacing threshold, 99% His-bundle pacing, and <1% right ventricular apical pacing. There was also no evidence of lead dislodgement or recovered atrioventricular nodal conduction.

**Table 1:** Baseline Characteristics of the 10 Patients Included in the Case Series

Mean age	75.4 years
Male sex	3 (30%)
Body mass index	31.7 kg/m^2^
Mean left ventricular ejection fraction	55%
CHA_2_DS_2_-VASc score, points	4
Hypertension	9 (90%)
Diabetes mellitus	4 (40%)
Coronary artery disease	5 (50%)
Congestive heart failure	5 (50%)
Chronic kidney disease	3 (30%)
Baseline conduction system disease	1 (10%)
Valvular heart disease	5 (50%)

### Operative technique

**Figure 1:**
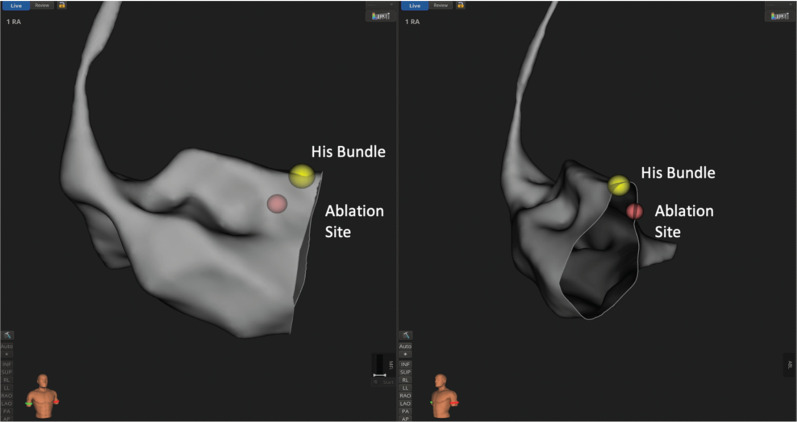
A three-dimensional electroanatomic map showing the His bundle and successful atrioventricular node ablation site.

**Figure 2:**
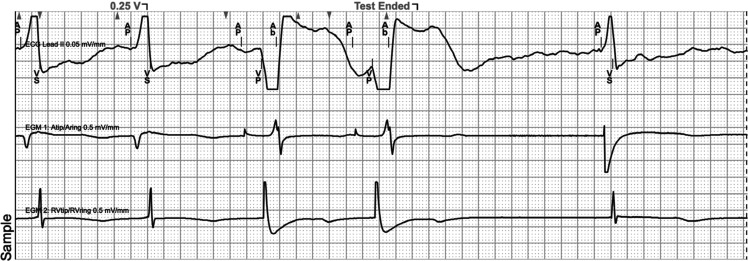
Pacemaker tracing during His-bundle lead threshold testing. The first two beats show successful His-bundle capture followed by appropriate inhibition of right ventricular apical pacing. The third and fourth beats show successful right ventricular apical pacing after loss of His-bundle capture.

**Figure 3:**
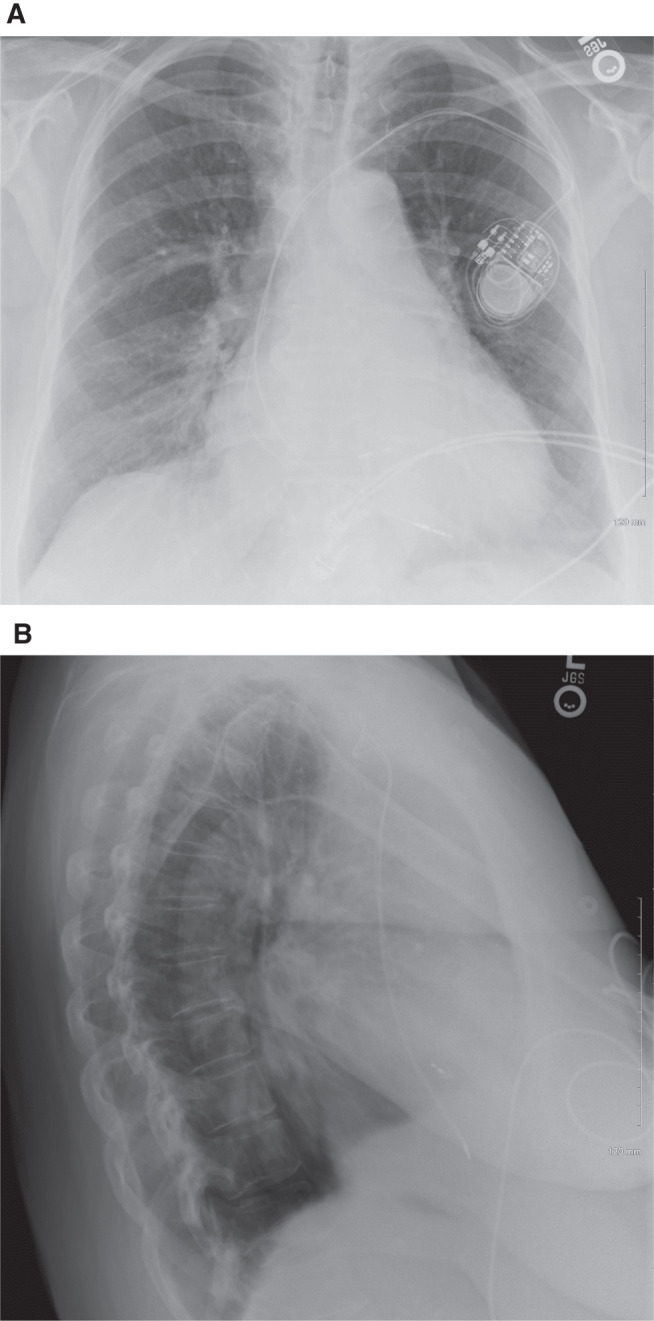
Postero-anterior **(A)** and lateral **(B)** postoperative chest X-rays showing satisfactory lead positioning.

**Figure 4:**
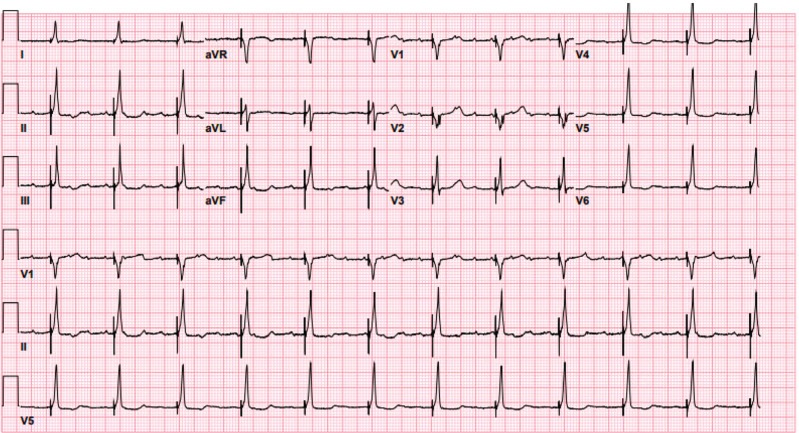
A 12-lead electrocardiogram following successful ablation of the atrioventricular node and pacemaker implantation showing His-bundle pacing.

Using an axillary approach with a micropuncture needle set, two introducer wires were placed. We initially proceeded with the placement of a pacing lead in the right ventricular apex via the usual implantation technique. We subsequently placed a deflectable sheath over the second introducer wire through which an ablation catheter was advanced into the right atrium, and a three-dimensional (3D) electroanatomic map of the right atrium, His-bundle region, and right ventricle was created. The ablation catheter was subsequently placed into the His-bundle region as guided by the electrograms and electroanatomic map. It was then withdrawn inferiorly and posteriorly into the region of the atrioventricular node with an atrial:ventricular electrogram ratio of 0.5:1 **(Figure 1)**. The right ventricular apical lead was programmed to pace at 40 pulses per minute. Ablation was then performed with the achievement of complete heart block. We then waited for at least 5 min to confirm the absence of residual atrioventricular conduction. The ablation catheter and deflectable sheath were then removed and exchanged for a His sheath. A His-bundle lead was then placed in the usual fashion with the attainment of selective His capture, if possible. The His-bundle lead was then connected to the atrial port of the pacing device, and the right ventricular apical lead was connected to the ventricular port. The pacing mode was programmed to DDDR and, in Medtronic devices, the ventricular safety pacing feature was turned off to prevent competitive right ventricular apical pacing. Prior to closure, the captured QRS after His pacing in the atrial channel was checked for appropriate sensing on the ventricular channel, followed by inhibition of right ventricular apical pacing. Threshold testing was then performed to ensure appropriate right ventricular apical pacing after loss of His capture **(Figure 2)**. If the device parameters were clinically acceptable, the pocket was irrigated and then closed in the usual fashion. The device parameters were longitudinally rechecked remotely and in-person indefinitely. A two-view chest X-ray **(Figure 3)** and a 12-lead electrocardiogram **(Figure 4)** were obtained postoperatively.

## Discussion

In recent years, an exciting new frontier of conduction system pacing has emerged, using the most theoretically bio-efficient way to pace the heart, employing the native conduction system. This pragmatic approach has proven itself in clinical trials, and several papers have demonstrated its tangible benefits with promising clinical outcomes.

Although right ventricular pacing has long-established benefits in patients with severe conduction disease, it does not come without significant risks, including dyssynchrony causing mitral and tricuspid regurgitation,^1^ decreased myocardial perfursion,^2^ increased risk for atrial fibrillation and pacemaker syndrome, and ultimately an increased burden of heart failure hospitalization and ventricular arrhythmias as demonstrated most notably in the Dual Chamber and VVI Implantable Defibrillator (DAVID)^3^ and Mode Selection Trial (MOST)^4^ studies.

Although conduction system pacing techniques are not new, with open-chest His-bundle pacing in dogs reported as early as the 1960s^5^ and a successful human case series describing permanent His-bundle pacing reported as early as the year 2000, operative and technical challenges as well as limited long-term outcome data have slowed widespread adoption.^6^

The benefits of conduction system pacing are inherently due to the recruitment of the native conduction system, avoiding dyssynchronous myocardial pacing. With heightened awareness regarding the potentially deleterious effects of right ventricular pacing in recent years and advancements of technology making His lead implantation more operatively feasible, attention has increasingly shifted toward targeting conduction system pacing in efforts to offset those known negatives of direct myocardial pacing in select patient groups.

Several trials have demonstrated the clinical superiority of His-bundle pacing over traditional right ventricular pacing. Abdelrahman et al., in their study of 332 patients, showed a significant reduction in the primary endpoint of death, heart failure hospitalization, or upgrade to biventricular pacing compared to right ventricular pacing (hazard ratio, 0.71; 95% confidence interval, 0.534–0.944; *P* = .02). At the 5-year follow-up, the rate of death or heart failure hospitalization was lower in their His-bundle pacing group relative to the right ventricular pacing group.^7^

Ideal intraoperative His lead placement is likely critical to driving short- and longer-term benefits. A well-targeted placement location of the His lead should ideally result in selective His lead capture with an isoelectric interval between the pacing spike and QRS. Non-selective His capture is expected to show a pseudo-delta appearance, which reflects septal capture prior to the His capture and further propagation via the intrinsic conduction system. The benefits of conduction system pacing are not only limited to promoting electrical and mechanical synchrony but can also correct bundle branch block in the majority of those with proximal His block location.^8,9^

In this case series, we set out to demonstrate the feasibility of combining two procedures into one: 3D electroanatomic mapping to locate the His-bundle region and ablate the atrioventricular node followed by His-bundle pacemaker lead insertion using sole axillary access. This achieves multiple goals, including decreasing the likelihood of access site bleeding, cross-contamination, and other complications by requiring only one access site and, specifically, avoiding femoral access, minimizing the risk of extending the length of stay. More importantly, this ensures conduction system pacing facilitated by 3D electroanatomic mapping in a patient likely to require frequent pacing with a reliable backup. A similar approach could be used in patients with a cardiac resynchronization therapy device by plugging the His-bundle lead into the left ventricular lead port and programming left ventricular pacing first.

As such, patients with long-standing persistent or permanent atrial fibrillation undergoing ablation of the atrioventricular node are anticipated to require a high degree of future pacing and thus were chosen as the ideal population for this case series.

With the emergence of left bundle branch area pacing, we have been able to overcome some of the limitations of His-bundle pacing. In fact, after completion of our series, we applied the same principles described earlier but implanted the “His-bundle” lead in the left bundle branch area with excellent pacing parameters. As this technology continues to evolve and prove its safety and efficacy, we could contemplate the use of a single-chamber, left bundle branch area pacemaker with ablation of the atrioventricular node using an axillary approach. In this scenario, we would place two introducer wires and start by lead implantation followed by ablation of the atrioventricular node. The ablation catheter would then be removed and hemostasis obtained by manual compression.

### Limitations

Although the benefits of His-bundle pacing are numerous, there are several limitations and operational challenges with questions over long-term stability and loss of capture given the minute location of the His bundle, and, thus, the placement of a backup right ventricular lead in case of a loss of His-bundle lead capture is mandatory. Although the patients in our series have maintained a high level of lead stability, this does not necessarily guarantee long-term future lead performance. Another limitation of His-bundle pacing is that the current dual-chamber devices on the market do not possess a pre-specified “His port”; this can be bypassed by connecting the His-bundle lead to the atrial port, which is an innovative approach. However, care must be taken to properly identify the His-bundle lead with a note within the device settings, so as to avoid confusion if the patient falls under the care of a new provider who could mistakenly assume the His-bundle lead for a misplaced atrial lead, either from chest imaging or device interrogation.

Atrial oversensing, ventricular undersensing, failure to overcome distal conduction system disease, and premature battery depletion due to high-output pacing remain significant limitations to His-bundle pacing.^10^

## Conclusion

Implantation of a conduction system pacemaker and ablation of the atrioventricular node in the same setting using an axillary approach is feasible. The use of a right ventricular apical pacing lead and a dual-chamber generator provides a safe and reliable backup in case of a His lead failure.
